# Golden GATEway Cloning – A Combinatorial Approach to Generate Fusion and Recombination Constructs

**DOI:** 10.1371/journal.pone.0076117

**Published:** 2013-10-07

**Authors:** Stephan Kirchmaier, Katharina Lust, Joachim Wittbrodt

**Affiliations:** Centre for Organismal Studies, University of Heidelberg, Heidelberg, Baden-Württemberg, Germany; Institute of Molecular and Cell Biology, Singapore

## Abstract

The design and generation of DNA constructs is among the necessary but generally tedious tasks for molecular biologists and, typically, the cloning strategy is restricted by available restriction sites. However, increasingly sophisticated experiments require increasingly complex DNA constructs, with an intricacy that exceeds what is achievable using standard cloning procedures. Many transgenes such as inducible gene cassettes or recombination elements consist of multiple components that often require precise in-frame fusions. Here, we present an efficient protocol that facilitates the generation of these complex constructs. The golden GATEway cloning approach presented here combines two established cloning methods, namely golden Gate cloning and Multisite Gateway^TM^ cloning. This allows efficient and seamless assembly as well as reuse of predefined DNA elements. The golden Gate cloning procedure follows clear and simple design rules and allows the assembly of multiple fragments with different sizes into one open reading frame. The final product can be directly integrated into the widely used Multisite Gateway^TM^ cloning system, granting more flexibility when using a transgene in the context of multiple species. This adaptable and streamlined cloning procedure overcomes restrictions of “classical construct generation” and allows focusing on construct design.

## Introduction

Plasmid construction is a necessary task and often a serious challenge for molecular biologists. Novel tools and features are constantly being developed, many of which are increasingly difficult to integrate into plasmids. Therefore, the establishment and use of novel techniques in molecular biology greatly depends on available cloning strategies. For example, fusion proteins with linkers of varying length can have different properties [1], arguing for an evaluation of a number of candidate linkers to find the optimal one for a specific application. Often, this requires the careful design of individual cloning strategies for all planned candidates, which is time-consuming and can be technically demanding. Moreover, this process is inherently inflexible to rapid prototyping and the continuous consideration of new results obtained with earlier candidates. This problem becomes particularly evident in experimental approaches that rely on small repetitive elements for targeted genetic recombination. Such techniques require DNA constructs that contain multiple and quasi-palindromic recombination elements, like loxP sites, together with larger open reading frames. One option for the construction of such DNA is the design of a large multiple cloning site (MCS). Fragments are then assembled via conventional restriction-ligation cloning in a step-wise manner (see for example 2). This is labor-intensive and in many cases does not allow a quick change of a specific DNA fragment in the final assembly. Our goal was to provide a framework for the rapid and flexible generation of these complex transgenesis constructs with a simple, modular design strategy.

The Golden GATEway cloning kit is a combinatorial approach that couples Golden Gate cloning with Multisite Gateway^TM^ cloning. Golden Gate cloning was established in 2008 [3] and has been widely used since (for example see 4–6). This cloning technique utilizes so-called type II restriction endonucleases. These enzymes cut outside their non-palindromic recognition sequence and the resulting overhangs can be almost freely chosen [3]. Since the recognition sequence is removed from the properly cut DNA fragment, recutting is not possible. Therefore, cutting and ligation of the DNA fragments occur in one tube simultaneously, which leads to the properly ligated plasmid becoming increasingly enriched over time. The principle of Golden Gate cloning has been shown to be highly efficient and very versatile. However, one major drawback is that the recognition sequence of the used type II restriction endonuclease must not be present in the final assembly. Although mutagenesis of the components can be demanding, this is usually not a problem for protein coding genes because of the degeneracy of the genetic code. This workaround, however, cannot be applied to non-coding DNA elements, like enhancers, promoters, or untranslated regions (UTRs). These need to be re-characterized after mutagenesis since it is in most cases unpredictable how the mutation will affect their function.

We combined the efficiency and versatility of the Golden Gate cloning approach with another method that allows sequence-independent cloning, namely Multisite Gateway^TM^ cloning. This approach relies on the recombination between specific att sites mediated via a commercially available enzyme set (LR clonase II Plus from Life Technologies). Multisite Gateway^TM^ cloning is widely used in many different fields and species-specific vector collections are distributed (for example see 7–9). The Tol2kit is widely used in the zebrafish community and provides a set of already characterized promoters, fluorescent proteins, tags and polyA sites [8]. Thus, our Golden GATEway approach enhances an established and widely used cloning pipeline.

## Results

### The basics of Golden GATEway cloning

**Figure 1:**
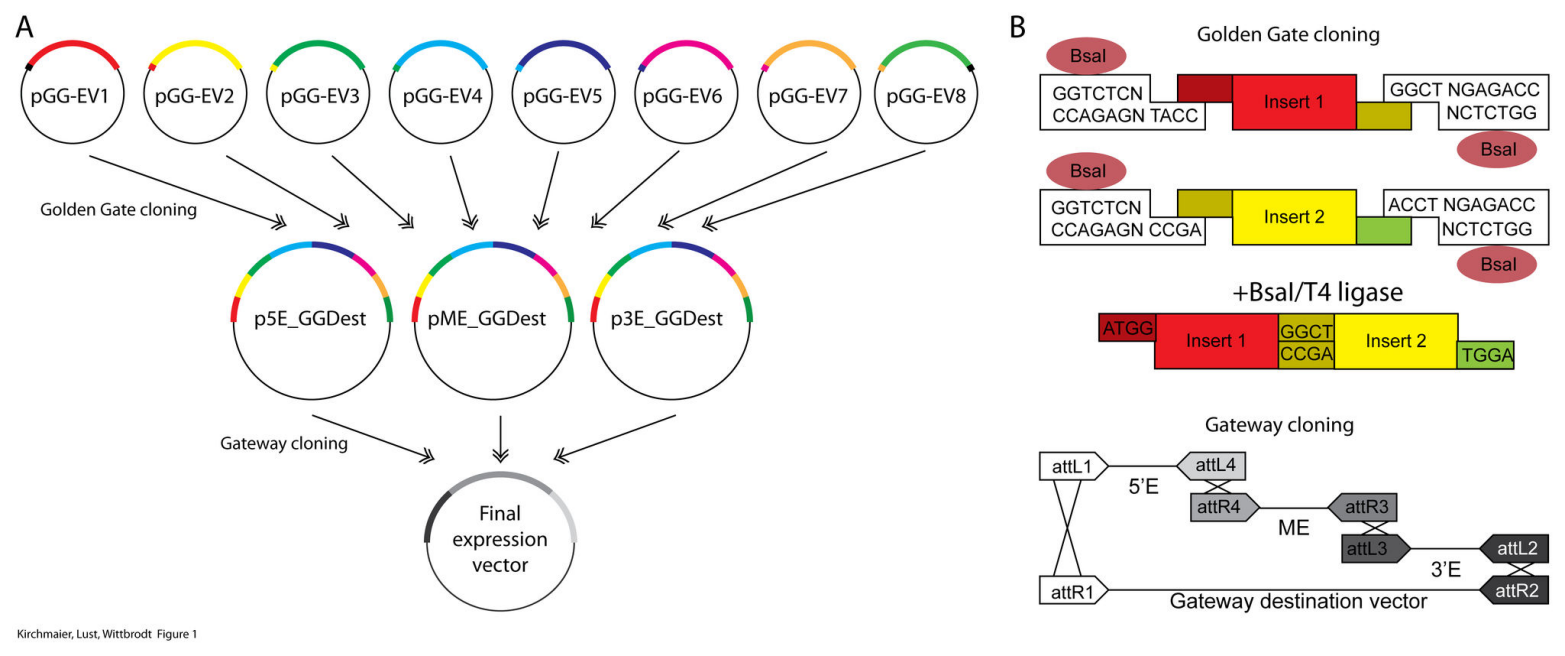
Summary of the Golden GATEway cloning kit. A) Outline of the entire cloning procedure. Eight different entry vectors (pGG-EVx) contain different inserts (colored bars). These inserts are assembled in a predefined order using a Golden Gate reaction into Gateway^TM^ entry vectors at any position (Threeway Gateway^TM^ cloning). These can then be assembled to establish a final expression vector in an LR reaction. Compatible overhangs are indicated on each Golden Gate entry vector. B) The principle of Golden Gate cloning is illustrated in the top scheme; the principle of Gateway cloning is illustrated in the bottom scheme. Golden Gate cloning utilizes type II restriction endonucleases to generate compatible overhangs that can be ligated with T4 ligase. The ligation of the two compatible inserts from the entry vectors one and two is illustrated. Gateway cloning relies on recombination of specific att sites using a commercially available enzyme mix (LR Clonase II, Life Technologies).

Our Golden GATEway cloning kit is characterized by the assembly of predefined DNA building blocks in two distinct steps (Figure 1). In the first step, Golden Gate cloning is used to assemble DNA fragments from Golden Gate entry vectors into predefined destination vectors in a given order. In the second step, these destination vectors are themselves entry vectors for Multisite Gateway^TM^ cloning to generate final transgenesis constructs. Thus, Golden Gate cloning can be used for the efficient construction of complex DNA elements. Subsequently, these elements seamlessly integrate with already widely established Gateway cloning components.

**Figure 2:**
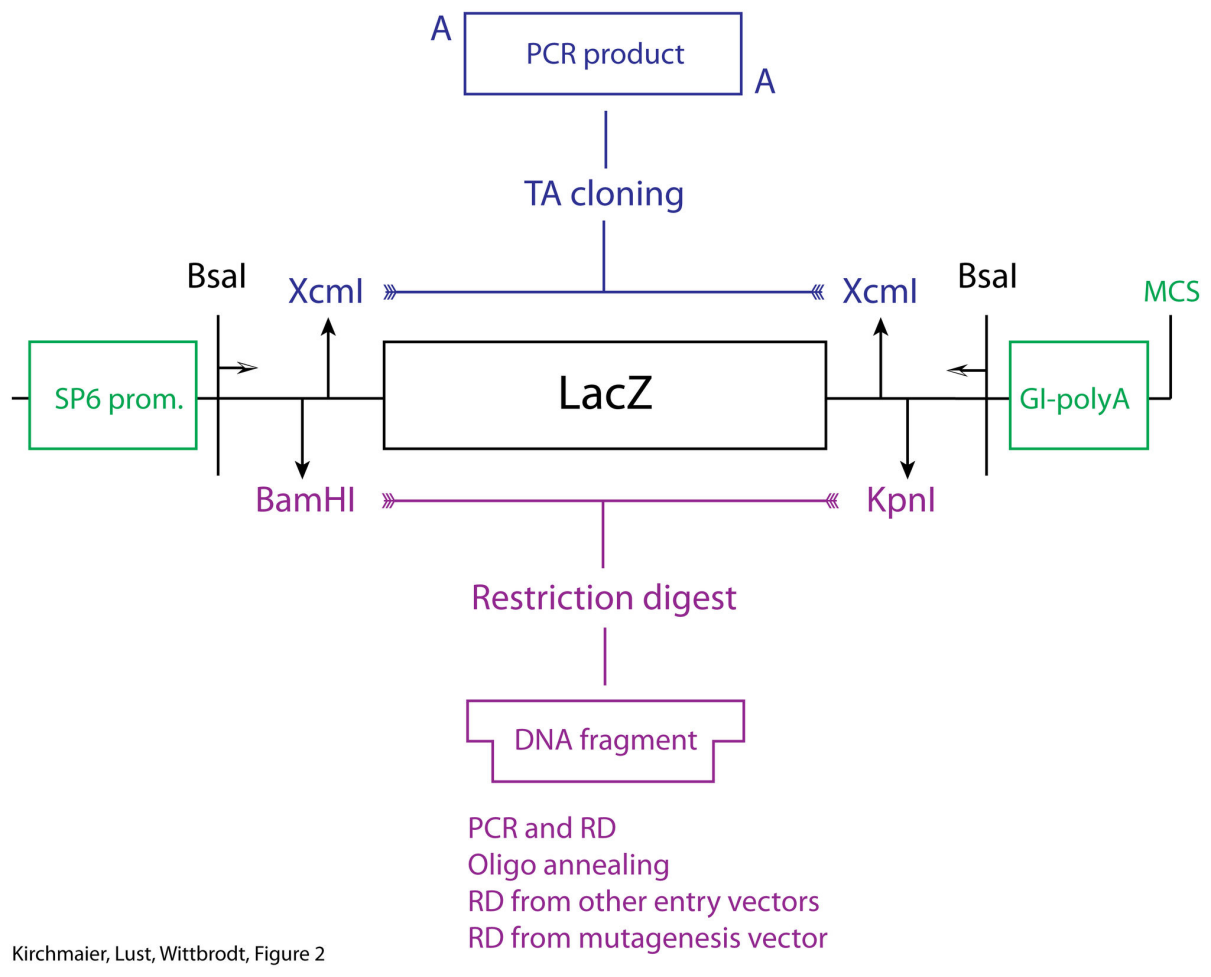
Golden Gate entry vector design and cloning. Cloning cassette used to fill Golden Gate entry vectors. Inserts can be introduced in two ways. XcmI restriction digest generates overhangs that can be used for TA cloning. Second, BamHI and KpnI sites can be used to clone inserts with different length either via standard ligation or an oligo annealing and cloning procedure. This cassette is flanked with BsaI sites used for the Golden Gate assembly. Each entry vector contains an SP6 promoter and a globin intron and SV40 polyA site flanking the insert for the generation of mRNA.

For the Golden Gate cloning step, we use up to eight different entry vectors per reaction. For most applications the assembly of eight fragments is enough, especially since it can be extended threefold in the subsequent Multisite Gateway^TM^ cloning step. These entry vectors represent eight consecutive positions in the assembled DNA element. The positions are defined via the overhangs that are created using BsaI restriction digest. All entry vectors share a cloning cassette that enables efficient cloning of fragments into the entry vectors (Figure 2). Additionally, this cloning cassette facilitates shuffling of DNA fragments between different entry vectors. The cassette consists of a LacZ gene for blue-white selection, flanked by a BamHI site at the 5’ end and a KpnI site at the 3’ end. These sites can be used for standard restriction ligation cloning as well as for the insertion of annealed oligos. In order to facilitate the cloning of PCR products using TA-cloning, XcmI restriction sites have been included. The overhangs from the BsaI restriction enzyme as well as the restriction sites for cloning are retained in the final assembly. Thus, a linker sequence is automatically introduced in the assembled DNA element. In order to minimize potential interference, we used overhangs that translate to small linker sequences (Table 1).

**Table 1:** Summary of overhangs and amino acid linkers.

**Position**	**Entry vector**	**5’ overhang**	**3’ overhang**	**aa linker**
1	pGGEV+1	ATGG	GGCT	MG-GS/SGTA/GT-GS
	pGGEV-1	GTAT	GGCT	(no ATG) - TA/GT-GS
2	pGGEV-2	GGCT	ACCT	GS-GS/SGTA/GT-TS
	pGGEV-2’	GGCT	TAAG	GS-GS/SGTA/GT-*
3	pGGEV-3	ACCT	ACTA	TS-GS/SGTA/GT-TT
	pGGEV-3’	ACCT	TAAG	TS-GS/SGTA/GT-*
4	pGGEV-4	ACTA	CTGC	TT-GS/SGTA/GT-LL
	pGGEV-4’	ACTA	TAAG	TT-GS/SGTA/GT-*
5	pGGEV-5	CTGC	TCAG	LL-GS/SGTA/GT-SG
	pGGEV-5’	CTGC	TAAG	LL-GS/SGTA/GT-*
6	pGGEV-6	TCAG	AGTT	SG-GS/SGTA/GT-LS
	pGGEV-6’	TCAG	TAAG	SG-GS/SGTA/GT-*
7	pGGEV-7	AGTT	GCCG	LS-GS/SGTA/GT-AG
	pGGEV-7’	AGTT	TAAG	LS-GS/SGTA/GT-*
8	pGGEV-8’	GCCG	TAAG	AG-GS/SGTA/GT-*

The entry vectors differ only in the overhangs that are created by BsaI restriction digest. These overhangs define the position of the fragment in the final assembly. Defined amino acid linker sequences are retained, since the overhangs as well as parts of the subcloning toolbox are retained in the final assembly. GS/x/GT are linkers introduced by the BamHI, KpnI restriction sites. TA-Cloning via the XcmI sites introduces the SGTA linker. Note compatible overhangs in consecutive entry vectors.

DNA fragments at maximum eight different positions can be assembled in a defined order. For the positions one to seven, two basic entry vectors have been generated; only one entry vector exists for position eight. At position one, the 5’ overhang contains either a linker or a start codon. The linker is necessary in order to avoid recombination elements as for example loxP sites within an open reading frame. The 5’ overhang with the start codon can be used for the generation of fusion proteins. Accordingly, two different destination vectors exist with adjusted 5’ overhangs. The Golden Gate destination vector with the start codon contains an optimized Kozak sequence prior to the overhang for efficient initiation of protein translation [10]. The entry vectors for the positions two to seven differ in their respective 3’ overhang. Either, these vectors allow fusion with the insert of the entry vector of the next position or they fuse to the destination vector. This is necessary to also allow the assembly of less than eight different entry vectors. The 3’ overhang of the destination vector contains an in-frame stop codon. Thus, for the creation of a functional fusion protein it is possible to use start and stop codon free fragments throughout. From each entry vector, mRNA can be in vitro transcribed using SP6 polymerase. In addition, a human globin intron and a SV40 polyA sequence [10] is located 3’ of the insert.

In the second step of the Golden GATEway cloning procedure, the assembled elements are integrated into a final expression construct using Multisite Gateway^TM^ cloning, which has virtually no sequence restrictions. The use of Multisite Gateway^TM^ cloning for the final assembly of the expression construct ensures that there is no need to re-evaluate already characterized promoters, UTRs and other noncoding sequences that contain BsaI sites.

We prepared Golden Gate destination vectors that can be used in Multisite Gateway^TM^ cloning at any position. This approach allows the assembly of a maximum of 24 fragments (3 (Multisite Gateway^TM^) x 8(Golden Gate)). This provides sufficient flexibility for most cases. Where needed, the system can be extended by the generation of additional Golden Gate entry vectors with new defined overhangs.

### A nomenclature to facilitate the design of fusion proteins

We propose a nomenclature that makes it possible to identify the insert, its position within the system, the presence of start or stop codons and its orientation. With our Golden GATEway cloning kit, fragments of different length can be assembled at predefined positions with defined spacer sequences in between them. It is possible to standardize the entry vectors using only few design guidelines.

Standardization is especially important for the construction of open reading frames (ORFs), which require in-frame assembly. This is achieved by cloning inserts in the ORF1 that is defined by the BamHI and KpnI restriction sites (Figure 2). Also, the presence of a start or stop codon has to be indicated. For example, the fluorescent proteins we use for the cloning of the recombination constructs are full open reading frames, whereas the entry vectors for the generation of fusion proteins lack both, start and stop codon. Another aspect of standardization is the possibility to shuffle inserts to other positions within the Golden gate assembly via BamHI, KpnI restriction-ligation cloning. Finally, the direction of the insert is important. For example, recombination sites like loxP can be used in forward or reverse orientation and their interaction differs with respect to their orientation. Detailed nomenclature guidelines are presented in table 2.

**Table 2:** Basic nomenclature rules.

**Basic nomenclature**	**pGGEV_Position_(+/-)Insert(+/-)_(+1/-1)_BK(+/-)/OA/TA(+/-)**
	*Description*
*pGGEV*	Identifier that indicates a Golden GATEway entry vector
*Position*	+/-1 … Entry vector at position one with(+) or without (-) an ATG
	2-7 … Entry vectors that allow fusion to the next position
	2’-8’ …Entry vectors that stop the assembly at a given position
*(+/-)Insert(+/-)*	Insert name with start (+) or stop (-) codon at the 5’ or 3’ end
*(+1/-1)*	Orientation of the insert: +1 … in frame with BamHI to KpnI
	Orientation of the insert: -1 … in frame with KpnI to BamHI
*BK/OA/TA-(+/-)*	Cloning method: BK … via BamHI, KpnI
	Cloning method: OA … via oligo annealing
	Cloning method: TA … via TA cloning using the XcmI sites
	(+/-) … reshuffling via BamHI, KpnI to another entry vector possible
*Examples*	pGGEV_+1_-Flag-_+1_OA-
	pGGEV_2_-hCre-_+1_BK+
	pGGEV_2_+nls-eGFP-Flag+_+1_BK+

The described nomenclature contains all necessary information to use the entry vectors for an assembly without the need to analyze the exact sequence. These rules are especially important for the generation of fusion proteins.

### Generation of Templates with User-Defined Multiple Cloning Sites

**Figure 3:**
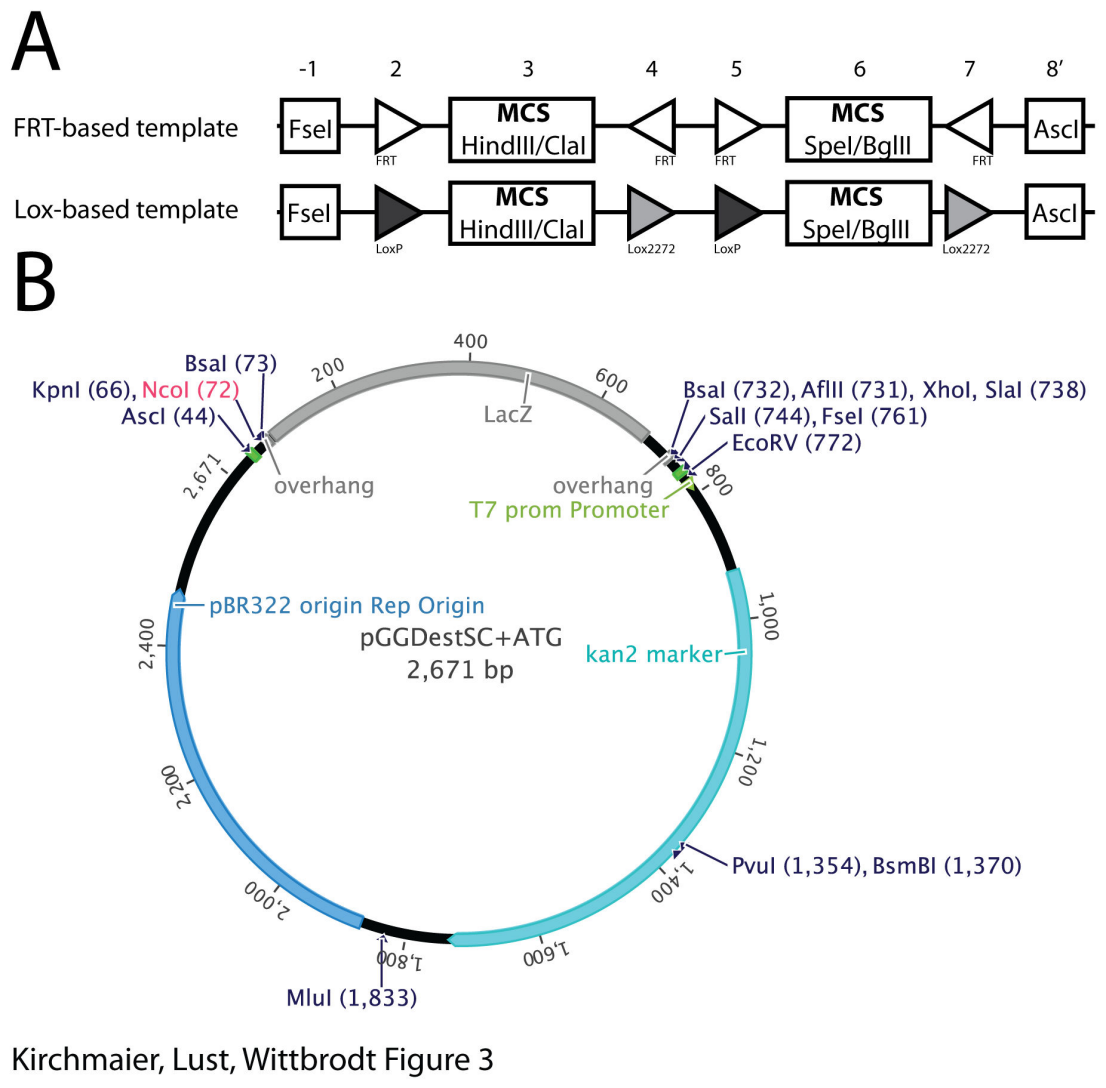
Generation of recombination templates using Golden Gate cloning. A) Schematic depiction of the generated recombination templates. The two recombination templates are based either on FRT or Lox elements in defined orientations. We included specific multiple cloning sites in the entry vectors -1, 3, 6 and 8’. B) Vector map of a subcloning destination vector with the restriction sites for the most common restriction endonucleases. The NcoI site (highlighted in red) is not present in the vector that lacks the ATG; otherwise restriction sites in all the vectors are identical.

In many labs, genes of interest are already sub-cloned into vectors that contain a predetermined multiple cloning site. Such constructs can be integrated into the Golden GATEway cloning kit with the generation of vector templates harboring specific restriction sites to insert already available genes of interests. This is especially interesting for recombination templates, i.e. DNA fragments that contain recombination elements in a defined order and orientation together with specific restriction sites for sub-cloning. Typically, the generation of such constructs involves the assembly of multiple small DNA fragments that are highly repetitive. Using the Golden Gate cloning step, we generated recombination templates using on the one hand only FRT sites and on the other hand loxP and lox2272 sites. These recombination templates are flanked with the 8-bp cutters AscI and FseI in the entry vectors one and eight, respectively. Additionally, specific restriction sites are inserted between the recombination elements (Figure 3A). We found that the majority of the tested clones are correct (FRT-based template seven out of eight /Lox-based template five out of eight) as verified by sequencing of the assemblies.

In order to facilitate subsequent cloning steps, we generated Golden Gate destination vectors, which contain the destination vector cassette flanked by AscI and FseI restriction sites. The remaining vector backbone harbors only few additional restriction sites, facilitating restriction-ligation cloning with newly assembled templates (Figure 3B).

### Using FlpO to illustrate site-directed mutagenesis

**Figure 4:**
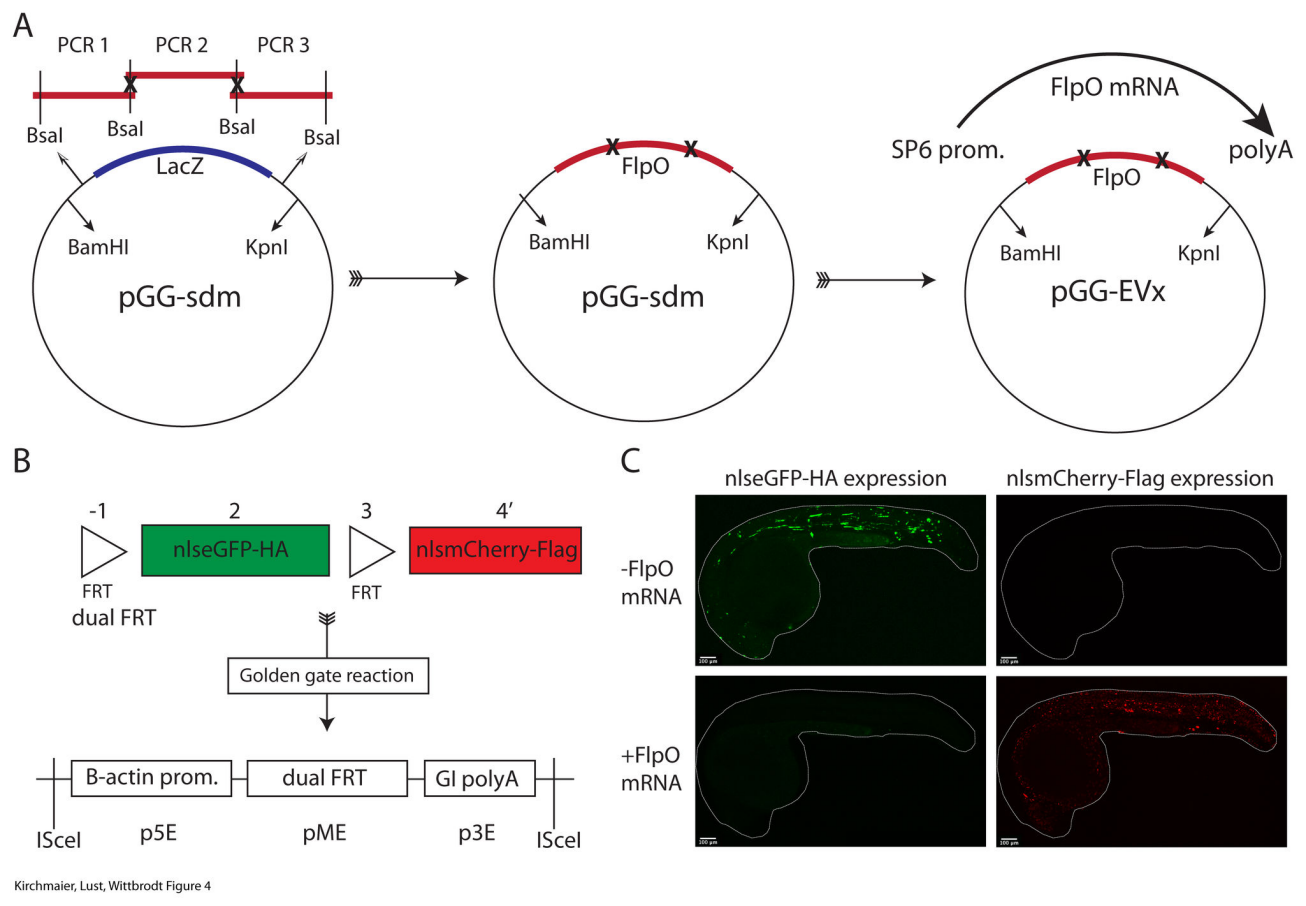
Golden Gate-based multisite mutagenesis. A) The FlpO ORF contains two internal BsaI sites. These are mutated by amplifying fragments of the FlpO ORF via PCR. The primers contain flanking BsaI sites that lead to compatible overhangs after restriction digest. The site-directed mutagenesis vector (pGG-sdm) contains BsaI sites for the mutagenesis assembly. Additionally, BamHI and KpnI sites allow the transfer of the mutated DNA assembly to Golden Gate entry vectors. From there, mRNA can be generated using SP6 RNA polymerase. Note that the pGG-sdm vector represents a standard Gateway^TM^ middle entry vector. B) A Golden Gate reaction is used to prepare a dual FRT-based recombination construct in the Gateway^TM^ middle entry vector. An LR reaction was prepared to generate a final expression construct. ISceI sites flank the expression cassette. The dual FRT element is driven by the zebrafish beta actin 2 promoter and a human globin intron with SV40 polyA is used in the 3’ entry vector. C) Zebrafish embryos were injected with the expression construct alone or in combination with FlpO mRNA. Without FlpO mRNA 64% (n=98) of the fish were positively injected and showed eGFP expression and the absence of mCherry. In combination with FlpO mRNA 60% (n=79) of the fish were positively injected and all of them showed the complete absence of green fluorescence and the appearance of red fluorescence, which is indicative for proper FlpO activity.

The Golden Gate procedure has one single sequence restriction: the recognition sequence of BsaI (GGTCTCN^NNNN) must not occur within the DNA fragments of interest. Its presence leads to re-cutting of the final assembly and thereby reduces the cloning efficiency. A PCR-based Golden Gate mutagenesis approach has been described previously [3]. We adapted this approach and established a vector that contains in-frame BamHI and KpnI restriction sites that flank specific BsaI restriction sites (Figure 4A). Specific overhangs defined by the primer sequences are generated with BsaI restriction digest of the PCR fragments. These overhangs then define the orientation and position of each PCR fragment in the assembly. This allows multisite mutagenesis with mutations in up to four base pairs at a single locus. Additionally, elements that need to be combined without linker sequences can be fused. We included the lacZ gene for colony selection. Importantly, since the cloning site is flanked by BamHI and KpnI restriction sites, the assembled DNA fragment can be easily transferred to any of the Golden Gate entry vectors. Additionally, the vector also represents a final middle entry vector for the Gateway cloning system. The procedure is illustrated with the FlpO open reading frame [11]. It contains two BsaI sites. We mutagenized them using the PCR-based mutagenesis approach. Five out of five tested clones carried mutated BsaI sites at the intended positions. We then cloned the mutated ORF into a Golden Gate entry vector using BamHI and KpnI (Figure 4A).

In order to illustrate that the mutated FlpO coding sequence as well as mRNA from the entry vectors is functional we generated a ubiquitously expressed dual FRT recombination construct with nuclear localized eGFP and mCherry using the Golden GATEway assembly method (Figure 3B). We co-injected the final expression construct into zebrafish embryos with and without FlpO mRNA (Figure 4C). Fish injected with the vector alone show only eGFP expression. Upon coinjection of FlpO mRNA with the vector, a switch of eGFP to mCherry expression is observed. This indicates that FlpO is functional and fully active in zebrafish embryos [12].

### Using Golden GATEway cloning to generate complex recombination constructs and fusion proteins

**Figure 5:**
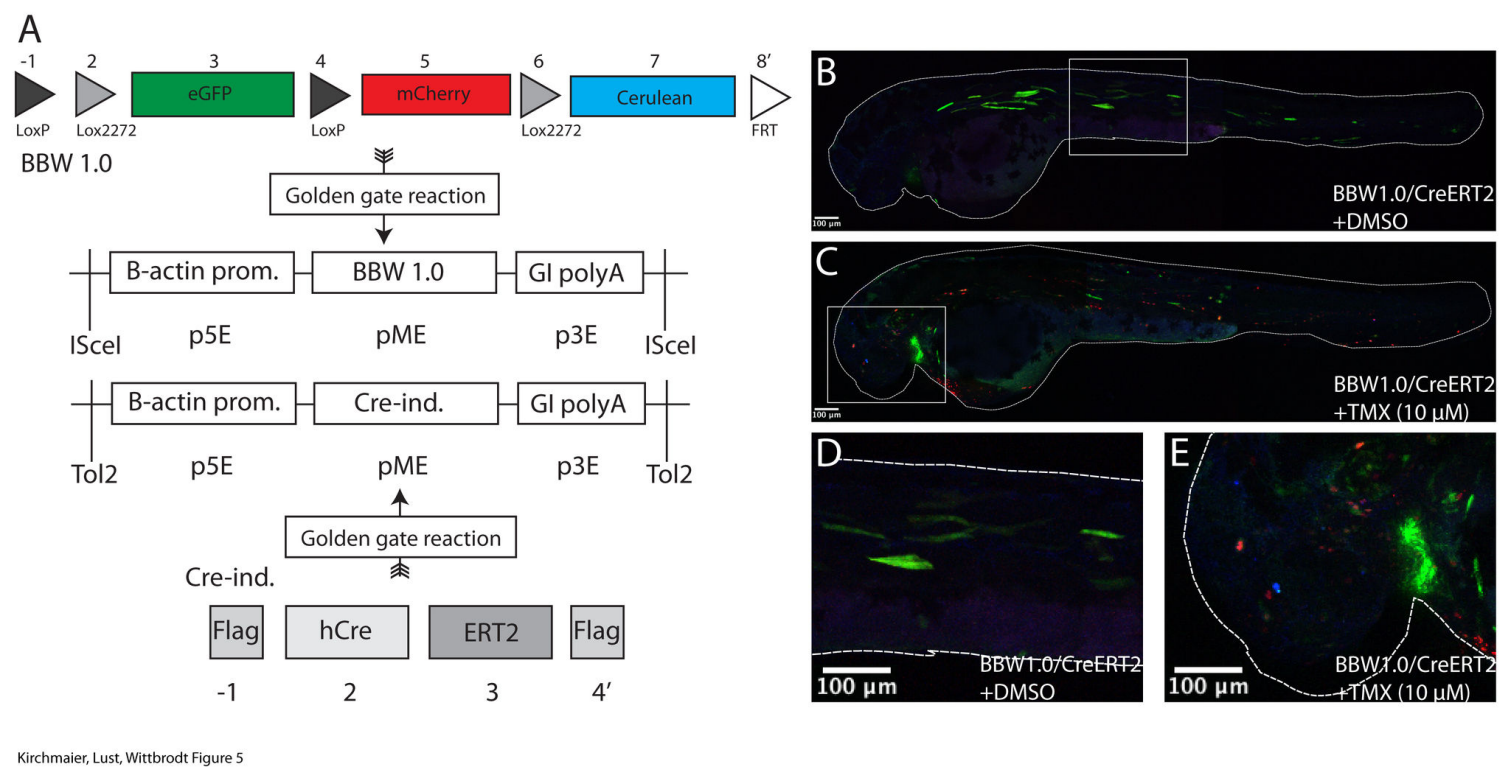
Complex recombination constructs and fusion proteins. A) A BBW1.0-like construct is assembled into the middle entry vector via a Golden Gate reaction 8 entry vectors are used that contain either recombination elements (LoxP, Lox2272 and FRT sites) or fluorophores. Additionally, the ORF that encodes for the tamoxifen-inducible Cre recombinase was generated with the tamoxifen-sensitive estrogen receptor (ERT2) and flanking Flag tags. The Golden Gate assemblies are made in the Gateway^TM^ middle entry vector. Subsequently, these are combined with the ubiquituous beta actin 2 promoter and the globin intron-SV40 polyA. B-E) Both vectors were coinjected with Tol2 mRNA. 57% (n=43) of the fish were transiently eGFP-expressing. Those were split in two groups and treated with either tamoxifen dissolved in DMSO or DMSO alone. All fish transiently injected with the BBW1.0 construct and CreERT2 retained green fluorescence after addition of DMSO. Addition of tamoxifen induced cell-specific recombination in all embryos.

We used the Golden Gateway cloning kit to build a brainbow 1.0 version [13] using the CreLox system (Figure 5A). For that we employed fluorescent proteins as final open reading frames in the entry vectors two (eGFP), five (mCherry) and seven (mCerulean). We combined them with loxP and lox2272 sites in the entry vectors one, two, four and six. Upon Cre activity, either the loxP sites or the lox2272 sites will recombine and the expression of fluorescent proteins will indicate where recombination has occurred (Figure 5B-E). No recombination is indicated by eGFP expression. Upon recombination between the loxP sites, mCherry is expressed and upon recombination between the lox2272 sites mCerulean is expressed. In order to remove potential tandem insertions, a forward FRT site at the 3’ end of the assembly (position eight) was included. We assembled this brainbow 1.0 construct in the Gateway middle entry vector and prepared a Multisite Gateway^TM^ reaction to generate an ISceI transgenesis vector that drives the brainbow cassette with the ubiquitous beta actin 2 promoter [8] and the globin intron SV40 polyA [10].

To generate a commonly used drug-inducible version of the Cre recombinase based on the ERT2 receptor [14] (Figure 5A), we fused the receptor to the Cre open reading frame. This allows nuclear entry upon tamoxifen treatment. We inserted Flag tags into the first and fourth entry vector. The second entry vector contained a Cre recombinase and the third entry vector was filled with a tamoxifen-inducible ERT2 fragment. This CreERT2 construct was assembled in the Gateway middle entry vector. Subsequently, for the expression construct, we used a Tol2 Gateway destination vector with the beta actin 2 promoter [8] and the globin intron SV40 polyA [10]. The brainbow 1.0 vector was co-injected with Tol2 mRNA [15] and the vector encoding inducible Cre into zebrafish embryos (Figure 5B-E). One day after injection dechorionated embryos were treated with tamoxifen. We detected mCherry and mCerulean expression after recombination in tamoxifen-treated fish while no recombination was apparent in DMSO-treated control fish. This experiment highlights that with our Golden GATEway cloning kit complex recombination constructs as well as fusion proteins are efficiently generated.

## Discussion

With our standardized cloning approach, the Golden GATEway cloning kit, we provide a framework that allows fast and efficient assembly of complex DNA constructs by combining Golden Gate cloning with Multisite Gateway^TM^ cloning. A unified nomenclature ensures that the design of the final expression vectors is flexible and simplified. The modularity of the system allows exchanging and reusing DNA fragments in multiple assemblies. We showed that recombination constructs as well as fusion proteins can be prepared efficiently and their functionality was demonstrated *in vivo* in zebrafish embryos.

The Golden GATEway cloning kit is especially designed for the generation of transgenesis constructs. Since the kit seamlessly integrates with standard Gateway^TM^ vectors, the final expression construct can be generated using already characterized elements. Importantly, the destination vector in the final assembly step determines the transgenesis method. Therefore, identical constructs can be used in different contexts and model organisms, as several species-specific Gateway^TM^ cloning kits have been established [7–9]. A full vector collection of the Golden GATEway kit will be made available to the community via Addgene.

## Materials and Methods

### Animals and ethics statement

All fish are maintained in the closed stocks at the University of Heidelberg. Zebrafish husbandry and experiments were performed according to local animal welfare standards (Tierschutzgesetz 111, Abs. 1, Nr. 1, Haltungserlaubnis) and in accordance with European Union animal welfare guidelines. The fish facility is under the supervision of the local representative of the animal welfare agency. Embryos of zebrafish (*Danio rerio*) of the wildtype strain AB/AB were used at stages prior to hatching. Zebrafish were raised and maintained as described previously [16].

### Generation of the vectors for the system

For generating the Golden Gate entry vectors a globin intron SV40 polyA [10] sequence (GIpolyA) was PCR amplified from the p3E-GIpolyA vector with forward primers containing an overhang with either BamHI or KpnI restriction sites as well as the two specific overhangs for each vector and the BsaI restriction sites. The CMV/SP6 polymerase site was PCR amplified from the p5E-CMV/SP6 vector (Tol2 Kit) [8] with the reverse primer containing the same overhang as used for the GIpolyA forward primer in reverse. The two fragments were fused by fusion PCR and blunt ligated into a PvuII digested pBS vector, in which the BsaI site in the Ampicillin gene was removed by site directed mutagenesis. The Lac operon was amplified from the pGem-T-Easy vector (Promega) with primers containing XcmI as well as BamHI and KpnI restriction sites. This fragment was cloned into the entry vectors via BamHI, KpnI restriction digest.

For generating the Golden Gate destination vector a multiple cloning site was amplified from the pME-MCS vector. The CMV/SP6 promoter was PCR amplified using a reverse primer with overhangs for the multiple cloning site, GIpolyA was PCR amplified with the forward primer containing an overhang for the multiple cloning site. After a triple fusion PCR the fragment was blunt ligated into the PvuII digested Middle entry vector of MultiSite Gateway^TM^ system (Invitrogen). After that the vector was cut with KpnI and XhoI and the lac Operon was inserted, which was PCR amplified from a pGem-T-Easy vector (Promega) using primers with overhangs containing the BsaI site and a restriction site for KpnI in the forward and XhoI in the reverse primer.

The destination vectors for site-directed mutagenesis were prepared by amplifying the lacZ operon with specific primers that contain BsaI as well as BamHI and KpnI sites. The product was eventually cloned into the Gateway^TM^ middle entry vector using the pENTR dTOPO cloning kit (Invitrogen).

### Generating new entry vectors

Entry vectors can be filled by standard BamHI, KpnI restriction/ligation cloning. For that, we used standard BamHI, KpnI enzymes from Thermo, Fisher together with the Fastdigest buffer (Thermo, Fisher). Vectors that can be used for TA cloning are prepared by XcmI restriction digest (Thermo, Fisher) and subsequent standard ligation with PCR fragments that contain A-overhangs. We adapted the oligo annealing and cloning procedure from Life Technologies. In short, oligos (obtained from Sigma or MWG Eurofins) were diluted in ddH_2_O to a final concentration of 200 µM. 5µl of each forward and reverse oligo, 2µl of 10x oligo annealing buffer (100 mM TrisHCL (pH 8); 10 mM EDTA (pH 8); 1 M NaCl) and 8µl of ddH_2_O were incubated at 95°C for 4 minutes and afterwards cooled at room temperature for 5-10 minutes. The 50µM annealed oligo mixture was diluted 100-fold in ddH_2_O. 1µl of annealed double-stranded oligo mixture was diluted in 5µl 10x oligo annealing buffer and 44µl ddH2O to obtain a final concentration of 10nM. The 10nM annealed double-stranded oligo dilution was used as an insert in a standard ligation reaction with T4 ligase (5U, Thermo, Fisher)

### Golden Gate Protocol

Reactions were set up using 20 fmol of each Golden Gate entry vector and the destination vector, 10 U of BsaI Restriction Enzyme (Thermo, Fisher Fast Digest Enzyme Eco31I) and 30 U of T4 DNA Ligase (Thermo, Fisher) in 2 µl 10x ligation buffer (10x ligation buffer was prepared by supplementing the Thermo, Fisher FastDigest buffer with 10mM dATP and 100mM DTT) to a final reaction volume of 20 µl with ddH2O. It is crucial for the reactions that the entry vectors are purified and diluted in pure water since buffer components might inhibit the Golden gate assembly. All reactions were incubated using the following cycling conditions: 2 min at 37 °C, 5 min at 20°C for 50 cycles, then 5 min at 50°C and 5 min at 80°C. 10µl of the reaction mix were used to transform chemical competent cells (Top10 strain from Life Technologies).

### Golden Gate Mutagenesis

Primers were designed with flanking BsaI sites. The flanking overhangs were chosen to ligate with the overhangs in the SdM-destination vector (Attachment to forward primer: CACC-*GGTCTC*-A-CGTG; attachment to reverse primer: ATTA-*GGTCTC*-G-CGAC). The four base pairs at the 5’ end are introduced so that BsaI can cut the linear PCR fragment. The internal overhangs were chosen so that BsaI sites in the FlpO ORF were removed with synonymous mutations. Subcloning/mutagenesis using that vector introduces one additional Valine at the 5’ and 3’ ends of the fragments next to the BamHI, KpnI sites. Subsequently, standard PCR reactions were made to amplify the FlpO fragments using Phusion polymerase (NEB). The fragments were purified and mixed with the SdM-destination vector in equimolar ratios (20fM). The Golden Gate cloning protocol described above was followed.

### Multisite Gateway^TM^ -LR Reactions

For LR reactions, we mix 0.5µl of each vector (20fmol) with 0.5µl of the LR Clonase II Plus (Invitrogen). The reactions were incubated at 25°C for 16 hours. After addition of 2µl water, the entire mix was transformed into 25µl OneShot Top10 or MachT1 cells (Invitrogen).

### Injection and drug treatment of zebrafish embryos

Zebrafish embryos were injected at the one-cell stage with either 7.5 ng/µl of DNA per vector or together with 5 ng/µl of RNA. At 24 hpf positively injected embryos were either used for imaging in the case of the FRT construct or for subsequent drug treatment in the case of the brainbow 1.0 construct. Tamoxifen (Sigma) was diluted in 100% DMSO and kept as a stock solution of 50 mM at -20°C.

For treatment of zebrafish embryos tamoxifen was used at a final concentration of 10 µM diluted in zebrafish medium. At 24 hpf embryos were dechorionated and induced for 8 hours in the dark at 25 °C. Control siblings were treated with corresponding dilutions of DMSO for the same time. Medium was replaced and embryos were imaged the next day.

### Imaging

Embryos were anesthetized with tricaine and embedded in 1% low melting agarose to acquire images using a Leica TCS SPE confocal microscope (Leica Microsystems, Germany). Stacked images were prepared using ImageJ.
